# Overexpression of PER2 Promotes De Novo Fatty Acid Synthesis, Fatty Acid Desaturation, and Triglyceride Accumulation in Bovine Mammary Epithelial Cells

**DOI:** 10.3390/ijms25189785

**Published:** 2024-09-10

**Authors:** Yifei Chen, Yujia Jing, Liangyu Hu, Zanna Xi, Zhiqi Lu, Juan J. Loor, Mengzhi Wang

**Affiliations:** 1 College of Animal Science and Technology, Yangzhou University, Yangzhou 225009, China; 2 Department of Animal Sciences, Division of Nutritional Sciences, University of Illinois, Urbana, IL 61801, USA

**Keywords:** period2, milk fat, transcription, translation, fatty acid

## Abstract

The core clock gene *Period2 (PER2)* is associated with mammary gland development and lipid synthesis in rodents and has recently been found to have a diurnal variation in the process of lactation, but has not yet been demonstrated in bovine mammary epithelial cells (BMECs). To explore the regulatory function of *PER2* on milk fat synthesis in bovine mammary epithelial cells, we initially assessed the expression of clock genes and milk fat metabolism genes for 24 h using real-time quantitative PCR and fitted the data to a cosine function curve. Subsequently, we overexpressed the *PER2* in BMECs using plasmid vector (pcDNA3.1-PER2), with empty vector pcDNA3.1-myc as the control. After transfecting BMECs for 48 h, we assessed the protein abundance related to milk fat synthesis by Western blot, the expression of genes coding for these proteins using real time-quantitative PCR, the production of triacylglycerol, and the fatty acid profile. The findings indicated that a total of nine clock genes (*PER1/2*, *CRY1/2*, *REV-ERBα*, *BMAL1*, *NCOR1*, *NR2F2*, *FBXW11*), seven fatty acid metabolism genes (*CD36*, *ACSS2*, *ACACA*, *SCD*, *FADS1*, *DGAT1*, *ADFP*), and six nuclear receptor-related genes (*INSIG1*, *SCAP*, *SREBF1*, *C/EBP*, *PPARG*, *LXR*) exhibited oscillation with a period close to 24 h in non-transfected BMECs (R^2^ ≥ 0.7). Compared to the control group (transfected with empty pcDNA3.1-myc), the triglyceride content significantly increased in the *PER2* overexpression group (*p* < 0.05). The lipogenic genes for fatty acid transport and triglyceride synthesis (*ACACA*, *SCD*, *LPIN1*, *DGAT1*, and *SREBF1*) were upregulated after *PER2* overexpression, along with the upregulation of related protein abundance (*p* < 0.05). The contents and ratios of palmitic acid (C16:0), oleic acid (C18:1n9c), and trans-oleic acid (C18:1n9t) were significantly increased in the overexpression group (*p* < 0.05). Overall, the data supported that *PER2* participated in the process of milk fat metabolism and is potentially involved in the de novo synthesis and desaturation of fatty acid in bovine mammary epithelial cells.

## 1. Introduction

The diurnal variation induces relatively stable and predictable environmental changes. Consequently, light-sensitive organisms have developed a circadian oscillation lasting approximately 24 h to adapt to this cyclic alteration [1,2]. The daily feeding pattern and milk synthesis pattern of dairy cows indicate a strong influence of the circadian system on the lactating process [3,4]. In general, dairy milk production exhibits diurnal variation with a peak during the day (0.1~0.65 kg higher than the nighttime level), while the percentage of milk protein and milk fat reaches its zenith at night (with 0.09% and 0.32% increments compared to daytime levels) [5]. It used to be assumed that milk production was primarily influenced by factors such as nutrition, season, environment, and other variables [2,6,7]. However, Alameen et al. [8] proposed that circadian rhythms are associated with milk yield and can serve as representative indicators of dairy cow metabolism and production performance. The circadian system, although not extensively elucidated, plays a crucial role in coordinating the metabolic and hormonal changes needed to initiate and sustain lactation [9]. And undoubtedly, disruptions in the circadian system can result in energy imbalances and lipid dysregulation, significantly impacting the lactating ability of rodents and cattle [10,11,12,13].

Period2 functions as a repressive element, exerting inhibitory effects on transcription activated by positive clock elements, thereby leading to the diurnal oscillation of genes [14]. Additionally, PER2 plays a role in regulating mammalian physiology. In comparison to pregnant mice, lactating mice exhibit an advancement of the *PER2* expression phase in mammary gland by 4 h, with the peak occurring at 18:00, showing a significant upregulation in expression level (2.5-fold); the ratio of CLOCK:PER2 protein in the breast is considerably higher than that in the liver [15]. The time-specific and tissue-specific expression pattern highlights the important function of clock proteins in mammary gland development and physiological adaptation during lactation. Additionally, mutant mice lacking functional *CLOCK* display impaired mammary clock activity and suffer from lactation defects despite normal virgin development [12]. *PER1*^−/−^ and *PER2*^−/−^ mice demonstrate impaired glucose tolerance [16]. These studies and lipidomics analysis provide evidence for the essential role of *PER2* gene in milk fat metabolism [17].

Milk fat synthesis is a complex and interactive process that involves the uptake of fatty acids from the bloodstream, the activation and intracellular transport of fatty acids, the de novo synthesis and desaturation of fatty acids, triglyceride (TAG) cycling, and the assembly and secretion of milk lipid droplets [18]. These processes are regulated by functional gene cascade controlled by transcription factors. Sterol regulatory element-binding protein (SREBP), located on the endoplasmic reticulum, plays a crucial role in regulating fatty acid metabolism [19] by directly activating the expression of more than 30 proteins, e.g., cluster of differentiation 36 (CD36) [20], acetyl-CoA carboxylase (ACC), fatty acid synthetase (FAS), insulin-induced gene 1 (INSIG1) [21], stearoyl-CoA desaturase (SCD) [22], etc. Circadian oscillators interact with these metabolic regulators to participate in various neuroendocrine signaling and metabolic pathways [23].

In light of the impact of *PER2* on metabolic functions and mammary gland development [12,24], we examined circadian changes in genes tied to milk fat synthesis and metabolism, pinpointing *PER2*’s role in triacylglycerol production and fatty acid profile in BMECs, to uncover the repressive arm of the circadian clock’s impact on bovine milk fat synthesis and metabolism.

## 2. Results

### 2.1. Gene Expression Pattern

**Figure 1:**
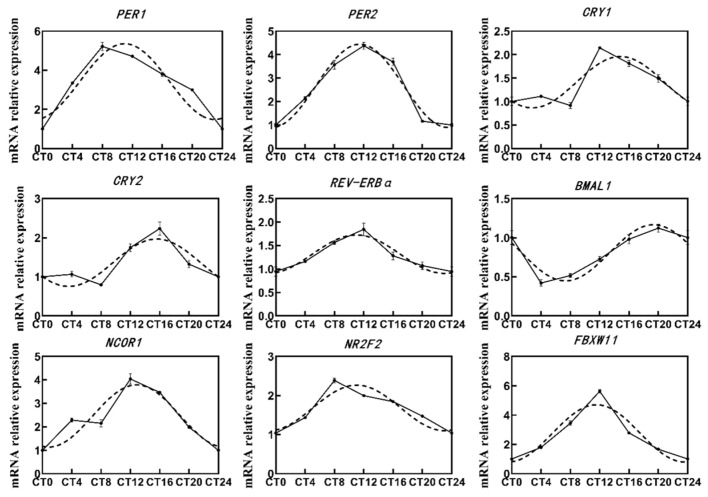
Temporal mRNA expression patterns of clock genes. The expression levels of clock genes within 24 h were subjected to nonlinear regression analysis (dashed lines, R^2^ ≥ 0.07, *p* < 0.05). Gene abundance was normalized using the geometrical means of GADPH, β-actin, and UXT.

Out of the 14 circadian genes, 9 were identified as oscillating in BMECs with *p* value < 0.05 (Figure 1). The transcripts of *RORα*, *CLOCK*, *CREB1*, *SIRT1*, *TIMELESS* were relatively constant throughout the day. The transcripts of *PER1*, *PER2*, *CRY1*, *CRY2*, *BMAL1*, *REV-ERBα*, *NCOR1*, *NR2F2*, and *FBXW11* accumulated in a circadian manner, albeit with a phase shift. The *PER1* and *PER2* peaked at circadian time 12 (CT12), while the *CRY1* and *CRY2* at CT16. *BMAL1* exhibited shallow circadian oscillations with zenith levels around CT20 and nadir levels around CT8. Genes involved in post-translational modification, e.g., *NCOR1*, *NR2F2*, and *FBXW11*, peaked around CT12.

**Figure 2:**
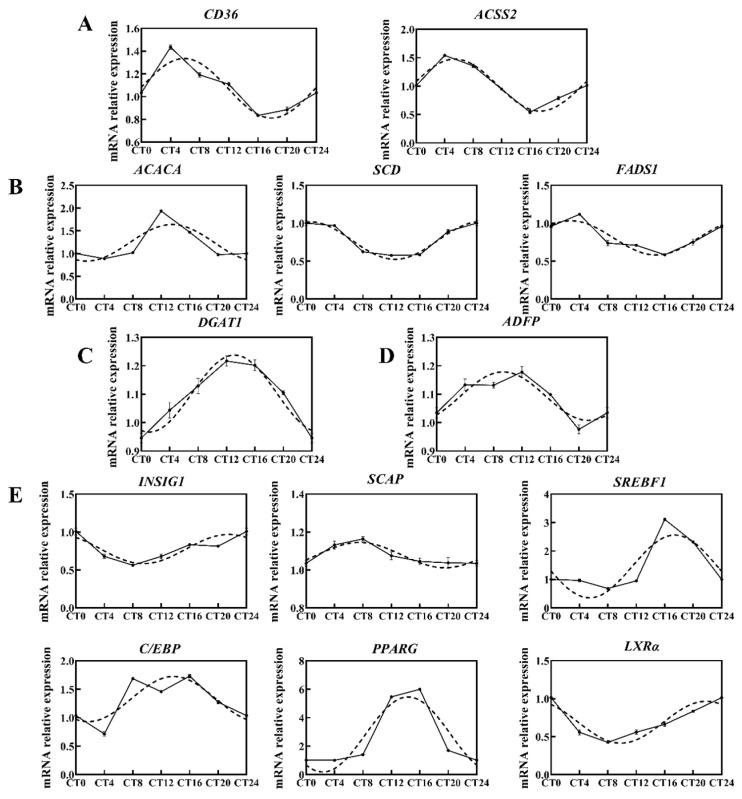
Temporal mRNA expression patterns of lipogenic genes. Target genes included those related to (**A**) FA uptake and activation (*CD36*, *ACSS2*), (**B**) de novo FA synthesis and desaturation (*ACACA*, *SCD*, *FADS1*), (**C**) triacylglycerol synthesis (*DGAT1*), (**D**) lipid droplet formation (*ADFP*), and (**E**) transcriptional regulation (*INSIG1*, *SCAP*, *SREBF1*, *C/EBP*, *PPARG*, *LXRα*). The expression levels of lipogenic genes within 24 h were subjected to nonlinear regression analysis (dashed lines, R^2^ ≥ 0.07, *p* < 0.05). Gene abundance was normalized using the geometrical mean of GADPH, β-actin, and UXT.

Genes involved in FA uptake (*CD36*, Figure 2A), FA activation (*ACSS2*, Figure 2A), de novo FA synthesis (*ACACA*, Figure 2B), FA desaturation (*SCD*, *FADS1*, Figure 2B), triacylglycerol synthesis (*DGAT1*, Figure 2C), lipid droplet formation (*ADFP*, Figure 2D), and nuclear receptor signaling (*SREBF1*, *PPARG*, *LXRα*, Figure 2E) were identified as diurnally oscillating in BMECs (*p* < 0.05). These findings demonstrated that the milk fat metabolism is expressed in a circadian manner.

### 2.2. Transfection Efficiency of PER2

**Figure 3:**
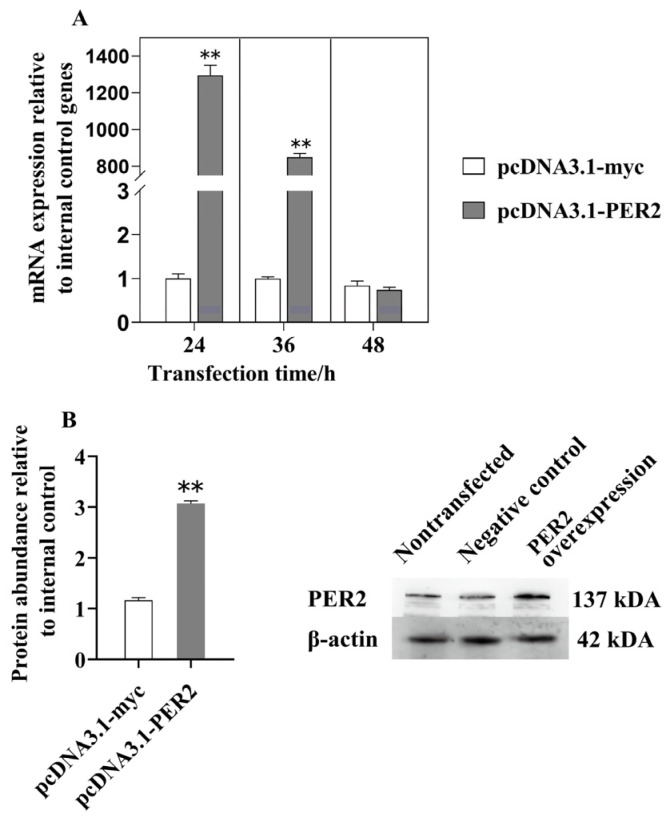
Overexpression of PER2 in BMECs. (**A**) qPCR analysis. Gene abundance was normalized using the geometrical mean of GADPH, β-actin, and UXT. (**B**) Western blot analysis was conducted to determine PER2 protein levels after 24 h of infection. Protein abundance was normalized with β-actin. The results are presented as LSM ± SE for three replicates. ** *p* < 0.01.

As shown in Figure 3A, the mRNA level of *PER2* was significantly elevated in BMECs transfected with pcDNA3.1-PER2 at 24 h and 36 h post-transfection, compared to those transfected with empty-pcDNA3.1. The peak value was observed at 24 h after overexpression, showing a remarkable increase of >1200-fold, which resulted in a more than 3-fold rise in the protein abundance of PER2 (*p* < 0.05, Figure 3B). Therefore, all experiments involving the overexpression of *PER2* were conducted using BMECs transfected with pcDNA3.1-PER2 for 24 h.

### 2.3. Lipogenic Genes mRNA Expression

**Figure 4:**
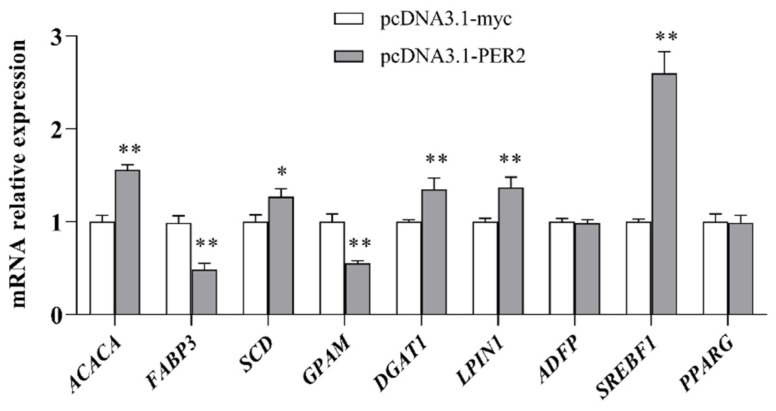
Effect of PER2 overexpression on the milk fat associated genes expression in BMECs. Values are reported as LSM ± SE for three replicates. * *p* < 0.05, ** *p* < 0.01.

Compared to control, *PER2* overexpression significantly enhanced the mRNA levels of several genes involved in fatty acid transport and triglyceride synthesis (*p* < 0.05, Figure 4), including acetyl-CoA carboxylase (*ACACA*, 1.69-fold), stearoyl-CoA desaturase (*SCD*, 1.33-fold), phosphatidic acid phosphohydrolase 1 (*LPIN1*, 1.37-fold), diacylglycerol acyltransferase 1 (*DGAT1*, 1.35-fold), and sterol regulatory element binding transcription factor 1 (*SREBF1*, 3.46-fold). No impact was observed on the mRNA levels of adipose differentiation-related protein (*ADFP*) and peroxisome proliferator activated receptor gamma (*PPARG*) compared to the control.

### 2.4. Protein Abundance and Milk Fat Synthesis

**Figure 5:**
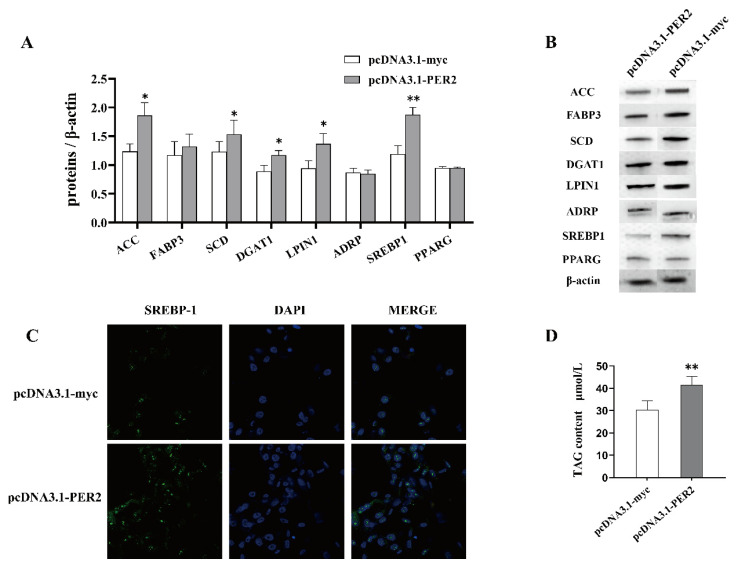
Effect of *PER2* overexpression on cellular triacylglycerol and protein content in key roles of synthesis in BMECs. (**A**) Protein levels were analyzed by Western blotting after 24 h of infection with the pcDNA3.1-PER2 plasmid. Values are expressed as LSM ± SE for three replicates. (**B**) Protein expression levels of milk fat synthesis-related proteins were determined by grayscale scan and presented as relative fold change compared to β-actin. (**C**) The cellular immunofluorescence staining was used to detect the nuclear localization of SREBP1, with the SREBP1 protein being stained with green fluorescence. Scale bar: 10 μm. (**D**) The amount of triacylglycerol in BMECs was quantified using a standard curve following the manufacturer’s protocols. Values are expressed as LSM ± SE for six replicates. * *p* < 0.05, ** *p* < 0.01.

The protein abundances involved in fatty acid de novo synthesis (ACC, 1.51-fold), fatty acid desaturation (SCD, 1.32-fold), triacylglycerol synthesis (LPIN1, 1.32-fold; DGAT1, 1.45-fold), and transcriptional integration (SREBP1, 1.57-fold) were improved by varying degrees (*p* < 0.05, Figure 5A). No impact was observed on the protein associated with fatty acid binding (FABP3), lipid droplet formation (ADRP), and peroxisome proliferator activated receptor (PPARG). As is shown in Figure 5C, PER2 overexpression increased SREBP1 translocation to the nucleus, and the fluorescence intensity in the nucleus was significantly higher than that in the control group. Additionally, there was an upregulation in the total cellular triacylglycerol content in cells transfected with pcDNA3.1-PER2 (*p* < 0.05, Figure 5D) compared to cells transfected with empty vector pcDNA3.1-myc.

### 2.5. Fatty Acid Content

In addition to an overall increase in TAG level, the transfection of pcDNA3.1-PER2 into BMECs also resulted in alterations to the cellular fatty acid composition (*p* < 0.05, Table 1), including increased proportions of hexadecenoic acid palmitic acid (C16:0), methyl trans-9-octadecenoate (C18:1n9t) and oleic acid (C18:1n9c), as well as decreased proportions of acetic acid, stearic acid (C18:0), and erucic acid (C22:1n9).

## 3. Discussion

Circadian rhythms control lipid metabolism pathways in major metabolic organs, with core circadian genes regulating the expression of numerous clock-controlled genes involved in lipid metabolism [25]. BMAL1 is highly recruited during adipocyte differentiation and lipogenesis [20], occupying thousands of DNA-binding sites rhythmically in mouse liver, including those involved in cholesterol and triglyceride metabolic pathways [26,27]. Genome-wide studies have shown that core clock protein DNA-binding sites are enriched in genes related to lipid metabolism, particularly triglyceride biosynthesis [28].

Genes involved in lipid metabolism are under the regulation of the circadian clock; however, lipids are not genetically encoded. Instead, they are synthesized by various metabolites and enzymes [29]. Examining gene expression alone is insufficient to fully interpret milk fat metabolism. Therefore, an increasing number of studies have implemented mass spectrometry-based approaches such as metabolomics for the large-scale analysis of metabolites and lipidomics for the targeted analysis of lipids [30]. These studies have revealed that different classes of lipids, including fatty acids, glycerolipids, glycerophospholipids, sphingolipids, and sterol lipids, exhibit circadian regulation [31,32]. The concentrations of ammonia and volatile fatty acid (VFA) in dairy cows’ rumen follow a well-defined daily pattern where ammonia peaks around 7:00 am while VFA peaks around 19:00 pm; dry matter, starch, and neutral detergent fiber reach their lowest level before feeding time [33]. The coordination between metabolism and circadian rhythm improves nutrient cycling efficiency, ensuring that storage molecules are not constructed or degraded simultaneously, thereby demonstrating potential benefits associated with time-based feeding management for dairy cows.

### 3.1. Circadian Oscillations of Genes

The diurnal oscillation of clock-controlled genes expression primarily manifests at the transcriptional level [34]. Intracellular signal transduction pathways play a crucial role in regulating the timing and entrainment of the biological clock system by modulating the expression of various clock genes [35]. To ascertain the expression pattern of the circadian clock genes in bovine mammary epithelial cell, we employed the cosine algorithm to fit the expression of related gene transcription and observed that 9 circadian clock genes and 13 lipogenic genes exhibited an almost 24 h oscillating expression pattern. Inevitably, the temporal resolution of circadian expression of some genes is limited by the 4 h sampling interval, potentially deviating from the actual dynamic changes in vivo.

Nuclear Receptor Subfamily 1 group D member (REV-ERBα) and Retinoic acid-related orphan receptor (ROR) play crucial roles as regulatory elements in the stabilization loop of the circadian clock. The *REV-ERB* can cause the recruitment of HDAC3 and deacetylate histones, in order to inhibit the transcription of target genes, such as *BMAL1* [36]. The *RORα* directly activates the transcription of *BMAL1* through two conserved ROR*α* response elements [37]. *REV-ERBα* may be more significant in maintaining clock stability compared to *RORα*, as we detected that *REV-ERBα* exhibited a shallow circadian oscillation in BMECs, while *RORα* mRNA level was relatively constant throughout the day. The peak of *REV-ERBα* occurred approximately 8 h earlier than that of *BMAL1*, potentially attributed to the daytime predominance of *REV-ERBα* protein expression and its preferential binding affinity towards *BMAL1* over *RO*Rα, which partially inhibited the transcriptional activity of *RORα* during daylight [38].

In addition to the transcriptional activators and inhibitors, the post-translational modifications and degradation of clock proteins, such as phosphorylation, ubiquitination, SUMOylation, acetylation, chromatin remodeling, etc., are also crucial steps in regulating the circadian cycle [36]. Histone deacetylase Sirtuin 1 (SIRT1) [7] and nuclear receptor corepressor 1 (NCOR1) [24] are transcription factors involved in the core clock histone deacetylation process, which promotes chromatin condensation and inhibits transcription. In our study, only *NCOR1* exhibited a high amplitude of 24 h diurnal oscillation with a phase similar to that of *PER*, peaking around CT12. *SIRT1* remained stable throughout the day, consistent with observations made by Asher et al. in mouse livers, possibly due to the post-transcriptional mechanisms, e.g., methylation, phosphorylation, and acetylation [39]. The F-Box and WD repeat domain containing 11 (FBXW11) protein inhibits the activity of cyclin dependent kinase 1 (CDK1), thereby promoting ubiquitination-mediated PER protein degradation by controlling DNA replication and repair [40]. The nuclear receptor NR2F2, a member of the steroid thyroid hormone-encoding nuclear receptor superfamily, directly activates the enhancer of the *Cyclin D1* gene and regulates the advancement of the cell division cycle from the G1 to S-phases [41]. The pronounced amplitudes of NR2F2 and FBXW11 provide evidence for the diurnal oscillations of post-transcriptional regulation.

The circadian clock exerts extensive influence on energy metabolism by regulating the rate-limiting steps in metabolic pathways [35]. Nuclear receptors are diurnally regulated in BMECs, hence linking the molecular clock with transcriptional networks involved in energy metabolism [42,43]. The mRNA levels of *SREBF* and downstream target genes were found to exhibit diurnal oscillation in the wild mice liver, with zenith levels around 22:00 and nadir levels around 10:00 [28]. We also detected similar responses in BMECs. Furthermore, the phase of *SREBF1* and *INSIG1* was approximately delayed by 4 h compared to *REV-ERBα*. This could be attributed to the early stage inhibition of *INSIG1* transcription by high level of *REV-ERBα*, resulting in the accumulation of nuclear SREBP. The high level of SREBP protein inhibited the transcription of *SCAP* and *SREBF*, which ultimately led to a delayed peak value of both *SREBF1* and *INSIG1* genes [6]. Adamovich hypothesized that *LPIN1* and *GPAT2* are direct target genes of clock genes in the liver, because the binding sites of core clock genes *BMAL1*, *CLOCK*, *PER*, and *CRY* are located at the same sites upstream of the *LPIN1* gene. And these clock genes exhibit different time phases: *BMAL* and *CLOCK* peak around 6:00, while *PER* and *CRY* peak around 18:00 [28]. However, a similar pattern was not observed in BMECs where *LPIN1* and *GPAM* expression remained relatively stable throughout the day. However, slight diurnal oscillations were observed in the expression of *CD36*, *ACSS2*, *ACACA*, *SCD*, *FADS1*, *DGAT1*, and *ADFP*. This discrepancy may be attributed to tissue-specific variations in core clock genes and metabolic gene expression patterns which are closely associated with specific tissue functions [3].

### 3.2. Fatty Acids Content

Diurnal variation in fatty acid synthesis is mediated partly by the effects of the circadian clock on SREBP1c and its downstream targets [35]. The rhythmic expression of *SREBF1* in the liver is independent of plasma triglyceride and glucose levels, potentially regulated by the liver clock [44], and plays a crucial role in integrating circadian and nutritional cues to regulate the diurnal oscillation of liver transcription [45]. Limiting feeding time for goats affects the expression of lipogenesis regulators (SREBP1c) and milk fat synthase (FASN and SCD1), resulting in the altered expression of mammary clock genes and diurnal changes in milk fat synthesis [46]. Therefore, we hypothesize that circadian clock effects on transcription factors, such as SPEBP1, may mediate diurnal variations in fatty acid synthesis through their downstream target genes [21,47]. Interestingly, our findings showed that *SREBF1* exhibited nearly 24 h diurnal oscillations in BMECs similar to *BMAL1*. This rhythmicity suggests a possible explanation for diurnal fluctuations in mammary lipid metabolism, while largely coordinating signaling pathways leading to this process [42]. In addition, the overexpression of *PER2* upregulated both mRNA expression (3.46-fold) and protein abundance (1.57-fold) for *SREBF1* but had no statistical effect on *PPARG* or the genes involved with lipid droplet secretion, which are mainly controlled by *PPARG* [48]. *PER2* may participate in milk fat synthesis through *SREBF1* rather than *PPARG*.

There are two primary sources of fatty acids in milk. The first is directly ingested from the diet through the blood, mainly consisting of long-chain fatty acids [49]. The second source is derived from de novo synthesis by mammary epithelial cell using acetic acid and beta-hydroxybutyric acid, which primarily consist of short- and medium-chain fatty acids [50]. Acetate and β-hydroxybutyrate are converted into acetyl-CoA and butyryl-CoA under the catalysis of acyl-CoA short chain synthetases (ACSS2) [51], which are then carbonylated to malonyl-CoA by acetyl-CoA carboxylase. These compounds combine with acetyl-CoA or a small amount of butyryl-CoA to saturated fatty acids under the action of fatty acid synthetase, with C16 being predominantly synthesized [18]. Our results show that compared to the control group, *PER2* overexpression significantly reduced acetic acid and increased C16:0, which corresponds to an increase in ACC mRNA and protein levels, suggesting that clock gene *PER2* plays a potential role in fatty acid de novo synthesis.

SCD and DGAT1 play a pivotal role in the triglyceride synthesis and impact the saturation of milk fat. Specifically, SCD primarily influences the production of medium- and long-chain monounsaturated fatty acids, while DGAT1 predominantly affects long-chain polyunsaturated fatty acids [18]. We observed an increase in saturated fatty acids (C16:0) and unsaturated fatty acids (C18:1n9t and C18:1n9c) following *PER2* overexpression. This could be attributed to enhanced transcription and translation level of ACC and SCD, along with the increased availability of substrates [52]. These findings demonstrate an augmentation in the de novo synthesis and desaturation of fatty acids within mammary epithelial cell after *PER2* overexpression. FABP3 serves as a specific transporter for long-chain fatty acids with high expression levels in the breast [53]. It is regulated by circadian clock (FABP3 increases by 100% when exposed to light under dark conditions) and is closely associated with the β-oxidation of fatty acids [54]. In dairy cows, the desaturation of palmitic acid and stearic acid by FABP3 may precede SCD [55]. Along with the aforementioned studies, we observed a decrease in *FABP3* mRNA expression following *PER2* overexpression.

### 3.3. Milk Fat Metabolism

Previous studies have demonstrated robust circadian rhythms of fatty acids in human and rat plasma, particularly in long-chain polyunsaturated fatty acids, which peak around 12:00, and triglycerides peak around 6:00. The circadian clock enhances TAG lipolysis in the morning, coinciding with the end of fasting/sleep and increased energy expenditure upon waking [56,57]. Adamovich [28] discovered that the accumulation of *LPIN1* is primarily clock-dependent and less affected by feeding time and proposed that *LPIN1* is a target gene in the transcriptional regulation of hepatic clock genes on lipid metabolism. Recent studies on goat mammary epithelial cells have demonstrated that LXR/SREBP1 and CREB1 can enhance the transcription of *AGPAT6*, *DGAT1*, *LPIN1*, and *GPAM* genes [58]. Conversely, the inhibition of *SREBF1* gene reduced the expression of *AGPAT6*, *LPIN1*, and *DGAT2* genes (by 23%, 28%, and 19%, respectively), as well as triglyceride content and milk lipid droplet accumulation in goat mammary epithelial cells [22]. In our experiment, we observed an increase in nuclear receptor (*SREBF1*) expression along with triglyceride synthesis-related genes (*LPIN1*, *DGAT1*) and proteins in BMECs after *PER2* overexpression, suggesting a potential regulatory effect of *PER2* on milk fat metabolism.

Adipose differentiation-related protein (ADRP) is a cytoplasmic lipid droplet binding protein responsible for encapsulation and secretion of lipid droplets [58]. *ADFP*, the gene encoded by ADRP, exhibits synergistic effects with Xanthine dehydrogenase (*XDH*) and Butyrophilin subfamily 1 member A1 (*BTN1A1*) in dairy cow mammary glands [18]. The overexpression of ADRP can enhance lipid accumulation and TAG concentration in GMECs [59]. However, no significant difference was observed between the control group and *PER2* overexpression group in ADRP mRNA level or protein abundance. This may be due to PER2 not affecting the lipid droplet formation.

The concentration of polyunsaturated fatty acids (PUFAs) in milk is known to be influenced by dietary factors and genetics. *FADS1* and *FADS2* code, respectively, for theΔ-5 and Δ-6 desaturases, rate-limit enzymes in the biosynthesis of PUFAs [60], which are essential for maintaining vital activities [61]. Our findings showed a shallow diurnal oscillation of nearly 24 h in mRNA expression of *FADS*; however, the content of ultra-long chain fatty acids (C20~C24) was low (only 5.12% to 12.11% of measured long-chain fatty acids). Furthermore, the content of polyunsaturated fatty acid (erucic acid, C22:1n9) decreased after *PER2* overexpression, suggesting that *PER2* may also affect the desaturation process through FADS for long-chain fatty acids [62]. The low content of long-chain fatty acids could be due to insufficient substrate supply or instability in mammary epithelial cell [49].

## 4. Materials and Methods

### 4.1. Cell Culture

Three mid-lactation Holstein cows (average 110 ± 5 days and 34.6 ± 0.5 kg/d of milk yield) were selected from the Yangzhou University experimental farm to obtain mammary gland tissue. Samples were obtained using a published mammary biopsy method [63] and washed with PBS, containing penicillin/streptomycin (100 IU/mL, Sigma-Aldrich, St. Louis, MO, USA). Primary BMECs were purified and cultured, according to our previously described protocol [64], in basal Dulbecco’s Modified Eagle Medium Nutrient Mixture-F12 medium (DMEM-F12, Gibco, Grand Island, NY, USA) supplemented with fetal bovine serum (10%, Gibco), prolactin (50 IU/mL, Gibco), insulin–transferrin–selenium (0.5 μg/mL, Gibco), cortisol (1 μg/mL, Gibco), epidermal growth factor (10 ng/mL, Gibco), penicillin–streptomycin (5 μg/mL, Solaribo, Beijing, China), amphotericin B (5 μg/mL, Solaribo), and gentamicin (5 μg/mL, Solaribo). Cells were cultured at 37 °C in a humidified atmosphere of 95% air and 5% CO_2_ with medium changes every 24 h. CT0/CT24 (circadian time) corresponded to the time when cells were first collected.

### 4.2. Eukaryotic Expression Plasmid Construction

The primers for the *PER2* gene were designed based on the complete coding sequence region of Bos Taurus *PER2* gene, which was previously published in GenBank (NM_001192317.1, https://www.ncbi.nlm.nih.gov/nucleotide/, accessed on 24 June 2024). The 5′ and 3′ ends of the primer were modified to include Hind III and EcoR I restriction enzyme cutting sites, respectively. Tsingke Biotech Co., Ltd. (Beijing, China), synthesized the primers with the following sequences: 5′-CAGCGGTTTAAACTTAAGCTTATGGATGGCTGCGCCGAC-3′ and antisense 5′-TGCTGGATATCTGCAGAATTCCTAGCGGACCTCACTGGC-3′.

Total RNA was extracted from tissue and cells using the TRIzol reagent (Tiangen Biotechnology Co., Ltd., Beijing, China), followed by reverse transcription into cDNA with the cDNA synthesis kit (TaKaRa Biotechnology Co., Ltd., Beijing, China). After the amplification of the cDNA, the 1-3876 nucleotide of *PER2* was amplified and subcloned into the shuttle vector pMD19-T on ice (pMD19-T 1 μL, PCR product 4 μL per 10 μL). The recombinant plasmid pMD19-T-PER2 was transformed into *Escherichia coli* DH5α competent cells and incubated on ice for 30 min, then subjected to a water bath at 42 °C for 90 s, followed by an ice bath for 2 min. Next, 890 μL LB liquid medium (added 10 g pancreatic peptone, 5 g yeast extract, and 10 g NaCl to 950 mL deionized water, adjusted to pH 7.0 using 5 M NaOH, diluted with deionized water to 1 L, and autoclaved) was added into the bacterial solution. The mixture was shaken at 37 °C for 1 h, then centrifuged at 1500× *g* for 10 min to collect the cells. The suspended cells were coated on LB solid medium (100 mL medium with 1.5 g agar powder, autoclaved for 15min, placed in a water bath at 55 °C, and then supplemented with 1% ambenzyl antibiotic), then blow-dried and incubated overnight at 37 °C in an inverted position. Several colonies were selected and inoculated into LB liquid medium, followed by overnight oscillation at 37 °C. Plasmids were extracted using a plasmid extraction kit (Tiangen Biotechnology Co., Ltd.) without endotoxin. Homologous recombination occurred between the shuttle vector and backbone vector, resulting in the positive recombinants of plasmid (pcDNA3.1-PER2) identified by kanamycin resistance and digestion with restriction endonucleases Kpn I and EcoR I. DNA sequence comparison was performed using SnapGene Viewer 5.2 (GSL Biotech LLC, Boston, MA, USA), and the results are presented in Figure S1.

### 4.3. Cell Transfection

The primary BMECs were cultured to passage 4 and then pooled. In accordance with Xiao et al. [65], the cells were synchronized using 100 nM dexamethasone (Gibco) in serum-free medium (DMEM-F12, Gibco) for 2 h before commencing the experiment. A total of 20 μL Lipofectamine 2000 (Invitrogen, Grand Island, NY, USA) was diluted with 500 μL reduced serum medium (Opti-MEM, Gibco) without antibiotics and incubated at room temperature for 5 min. And then each 6 μg plasmid was diluted with 500 μL reduced serum medium. The above two solutions were mixed and incubated at room temperature for 20 min. The BMECs in 60 mm Petri dishes (about 90% confluence) [66] were transfected with the pcDNA3.1-PER2 plasmid, with empty pcDNA3.1-myc as control. After transfection for 6 h, the complex medium was replaced with DMEM-F12 medium with bovine serum and antibiotics, for subsequent tests conducted at time points of 24, 36, and 48 h post transfection. qRT-PCR and Western blot were employed to determine the transfection efficiency and identify the optimal time point for transfection. Each treatment was performed in 3 replicates.

### 4.4. RNA Extraction and qRT-PCR

Total RNA was extracted from BMECs using the TRIzol reagent (Tiangen Biotechnology Co., Ltd.) following the manufacturer’s instructions. The concentration and integrity of RNA were determined by ND-1000 spectrophotometer (Nano-Drop Technologies, Wilmington, DE) and Agilent 2100 Bioanalyzer (Agilent Technologies, Santa Clara, CA, USA). Samples for subsequent analysis had an RNA Integrity Number (RIN) of ≥8.0. Primers were designed and verified using Primer Premier 5 (Premier Biosoft Interpairs, Palo Alto, CA, USA, Table S1).

The first-strand cDNA was synthesized (1 μg of RNA per 20 μL reaction) using the reverse transcription kit (TaKaRa Biotechnology Co., Ltd.) according to the manufacturer’s instructions. Real-time quantitative PCR (qRT-PCR) was performed using Universal SYBR qPCR Kit (Vazyme Biotechnology Co., Ltd., Nanjing, China). Reaction mixtures were preheated at 95 °C for 30 s, followed by a cycle of melting at 95 °C for 10 s and annealing at 60 °C for 30 s, repeated for a total of 40 cycles. The Biosystems 7500 Real-Time PCR System (USA) was utilized to determine the relative gene expression, and QuantStudio™ 7 Flex Real-Time PCR Software v2.3 (Applied Biosystems, CA, USA) was used to analysis data. The relative gene expression was calculated with the 2^−∆∆Ct^ method. The normalization of target gene abundance was performed by dividing their relative abundance of three housekeeping genes, i.e., glyceraldehyde-3-phosphate dehydrogenase (*GADPH*), β-actin (*ACTB*), and ubiquitously expressed prefoldin-like chaperone (*UXT*). The expression of the internal control genes was not affected by treatment (*p* > 0.05).

The diurnal expression of genes was fitted with the following equation, where *m* represents the average value of gene expression, *A* represents the magnitude of diurnal variation in genes, *x* is the conversion factor (i.e., *x* = 2π*t*/24) for day and nighttime *t* (24 h, cosine radian), *t* is the actual sampling time, and *w* represents the peak of gene expression:

*y*(*x*) = *m* + *A* × cos(*x* − *w*)

### 4.5. Protein Extraction and Western Blotting

The cells were washed twice with ice-cold PBS in 60 mm Petri dishes, followed by the extraction of total protein using RIPA lysis and extraction buffer (Thermo Fisher Scientific, Shanghai, China) supplemented with phenylmethanesulfonyl fluoride protease inhibitor (PMSF, Thermo Fisher Scientific) at a final concentration of 1 mM. The lysates were then centrifuged at 14,000× *g*, 4 °C for 10 min. Protein concentration was measured and normalized using the BCA protein assay kit (Thermo Fisher Scientific) according to the manufacturer’s instruction.

The proteins and 4× loading buffer (Thermo Fisher Scientific) were mixed in a ratio of 4:1 (wt:vol) and denatured at 95 °C for 10 min, followed by cooling at 4 °C for 5 min. Subsequently, the samples were subjected to separate protein using a 10% SDS/PAGE, and transferred onto a PVDF membrane (Bio-Rad, Hercules, CA, USA) using the Trans-Blot SD Semi-Dry Electrophoretic Transfer Cell (Bio-Rad) for 25 min. Prior to overnight incubation at 4 °C with primary antibodies for PER2, FABP3, ACC, SCD, LPIN1, DGAT1, ADRP, SREBP1, and PPARG (Zen-Bioscience Co., Ltd., Chengdu, China, Table S2), the membranes were blocked with Tris-buffered saline containing Tween-20 (TBST, 150 mM NaCl, 10 mM Tris-HCl, 0.1% Tween-20, pH 7.4) with 5% skim milk for 1.5 h. Horseradish peroxidase (HRP)-conjugated goat anti-mouse IgG and goat anti-rabbit IgG (Zen-Bioscience Co., Ltd.) were used as secondary antibodies. The visualization of membranes was conducted using the ChemiDoc MP System (Bio-Rad) with Clarity Western ECL Substrate (Bio-Rad) reagent. Band intensities were quantified utilizing Image Lab software (version 3.0, Bio-Rad). The intensity of ACTB was used to normalize target protein abundance.

### 4.6. Triacylglycerol Content Determination

The cellular TAG content in BMECs was quantified with the Tissue Triglyceride Assay Kit (Applygen, Beijing, China) following the manufacturer’s instructions. The procedure involved rinsing cells twice with PBS to remove glycerol, lysing at room temperature for 10 min (0.1 mL of lysate per 1 × 10^6^ cells), incubating at 70 °C for 10 min, and centrifuging at 447× *g* for 5 min to collect the supernatant. A total of 10 μL of supernatant was mixed with 190 μL of lysate, incubated at 37 °C for 15 min, and the reaction mixture was read on a microplate reader (SpectraMax M5, Molecular Devices, CA, USA). The TAG content in samples was determined by calculating against a standard curve according to the manufacturer’s protocols.

### 4.7. Immunocytofluorescence

Cells were seeded in confocal dishes and transfected as described above. Upon completion of the treatment, formalin-fixed cells were permeabilized with 0.1% Triton X-100 (Thermo Fisher Scientific) in TBS for 5–10 min and blocked with 3% BSA-PBS for 30 min at room temperature. Cells were probed with a SREBP1 polyclonal antibody (Thermo Fisher Scientific) in 3% BSA-PBS at a dilution of 1:100 and incubated overnight at 4 °C in a humidified chamber. Cells were washed with PBST and then labeled with goat anti-rabbit IgG conjugated with Alexa Fluor (Thermo Fisher Scientific) for 45 min at room temperature. DAPI (Thermo Fisher Scientific) was added and incubated for 5 min in the dark to stain the nuclei. The samples were observed under a laser confocal microscope (Perkin Elmer, Waltham, MA, USA).

### 4.8. Fatty Acid Measurements

Cells were scraped from 60mm Petri dishes using a cell scraper and centrifuged at 167× *g* for 5 min to discard the supernatant. The fatty acid mixed standards (Sigma) were utilized as the internal control. In accordance with Xu et al. [67], the cells were suspended in a mixture of sulfuric acid and methanol (sulfuric acid/methanol = 1:40) with a volume of 2 mL, transferred to a 15mL centrifuge tube with 200 μL of internal standard, ultrasonicated for 10min, and incubated in an 80 °C water bath for 1 h. After cooling the tube to room temperature, 2 mL 0.1 M hydrochloric acid and 800 μL hexyl hydride were added, vortexed for 30 s, and then centrifuged at 900× *g* for 5 min. The supernatant was transferred into a 2 mL silicified tube, added 0.5 g water-free sodium sulfate, shocked acutely, and centrifugated at 13,800× *g* for 3 min. The lipid supernatant was collected for fatty acid analysis by GC-MS (Thermo Fisher Trace GC ultraDSQII-AI3000) with a BP-5MS column (30 m × 0.25 mm × 0.25 μm) after being filtered through 0.45 μm organic membranes. The relative proportion of each fatty acid was determined by calculating its percentage of the total peak area that could be identified.

### 4.9. Statistics

Prior to statistical analysis, the normality of distribution was assessed using the Shapiro–Wilk test of SAS 9.4 (SAS Institute Inc., Cary, NC, USA). mRNA abundance data were log_2_-scale transformed to fit a normal distribution of residuals. The F test was employed to evaluate whether the amplitude of the cosine wave fitted to the data significantly exceeded zero. Nonlinear regression fitting requires R^2^ ≥ 0.7. Least square means and standard errors (LSM ± SE), were determined using the Lsmeans statement and means, and were compared using Tukey’s test when a significant interaction between pcDNA3.1-PER2 and empty pcDNA3.1-myc was observed. Statistical significance was defined as *p* < 0.05.

## 5. Conclusions

**Figure 6:**
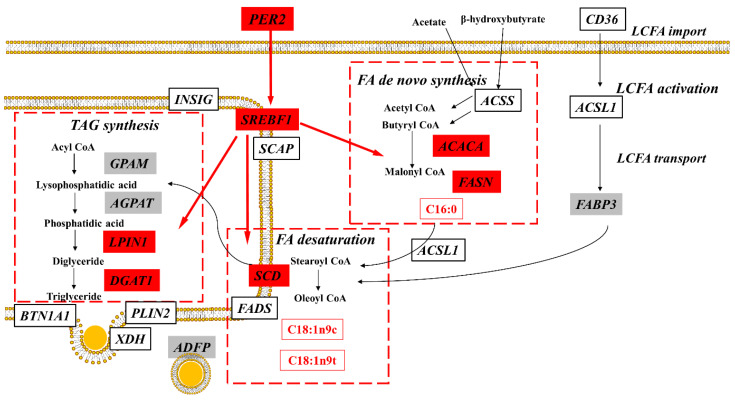
Effects of PER2 overexpression on milk fat synthesis in BMECs. The overexpression of PER2 significantly upregulated the expression of genes involved in de novo fatty acid synthesis, fatty acid desaturation, nuclear receptor and triglyceride synthesis (red shade = increase), while it had no impact on the expression of genes related to lipid droplet formation (grey shade = no change). White shades denote genes not measured in the present experiment.

Many genes involved in lipid metabolism are regulated by the molecular clock. A model summarizing the findings from the present experiment is reported in Figure 6. Our results strongly support the crucial role of PER2 in regulating the expression of genes coding for proteins involved in milk fat synthesis, particularly the de novo synthesis (*ACACA*) and desaturation (*SCD*) of fatty acid, as well as triacylglycerol synthesis (*DGAT1*, *LPIN1*), in bovine mammary epithelial cell. Additionally, the positive impact on the transcriptional integrator SREBP provides evidence for the interactive networks of transcriptional factors involved in controlling milk fat synthesis in bovines.

## Figures and Tables

**Table 1:** Effect of PER2 overexpression on the fatty acid content (μg/g) in BMECs.

Fatty Acid	pcDNA3.1-myc	pcDNA3.1-PER2	SEM	*p*-Value
C14:0	0.004	0.003	0.000	0.407
C16:0	0.022 ^a^	0.035 ^b^	0.003	0.014
C18:0	0.026 ^b^	0.017 ^a^	0.002	0.007
C18:1n9t	0.038 ^a^	0.052 ^b^	0.002	0.002
C18:1n9c	0.036 ^a^	0.045 ^b^	0.002	0.015
C18:2n6t	0.001	0.002	0.000	0.715
C22:1n9	0.009 ^b^	0.003 ^a^	0.001	0.000
C24:0	0.004	0.003	0.000	0.174
Acetic acid	17.703 ^b^	11.635 ^a^	1.279	0.009
Propionic acid	0.748	0.641	0.085	0.276
Isobutyric acid	0.664	0.714	0.093	0.613
Butyric acid	0.822	0.654	0.106	0.189
Isovaleric acid	0.250	0.263	0.081	0.881
Valeric acid	0.149	0.139	0.031	0.769

^a,b^ Within a row, values with no common superscripts differ significantly (*p* < 0.05). Mean values are based on three replicates.

## Data Availability

The data presented in this study are available on request from the corresponding author.

## Supplementary Materials

The following supporting information can be downloaded at: https://www.mdpi.com/article/10.3390/ijms25189785/s1.
