# Supplementary material for: Overexpression of PER2 Promotes De Novo Fatty Acid Synthesis, Fatty Acid Desaturation, and Triglyceride Accumulation in Bovine Mammary Epithelial Cells
Source: Int J Mol Sci. 2024 Sep 10;25(18):9785. doi: 10.3390/ijms25189785 (PMC11431620; doi:10.3390/ijms25189785)
Supplement: Supplementary file 1 [file ijms-25-09785-s001.zip › ijms-3171971-supplementary.pdf]

Table S1. Primers for the target genes are listed in the table

Gene	Primer sequence		Accession Number
PER1	GGGCAAGGACTCAGAAAGAAC	GAACTCCTGGTTGGCTTGC	NM_001289772.1
PER2	AGCGTGTTCCATAGCTCCAC	ATCTCGCTCTCGTGGCTTT	NM_001192317.1
CRY1	TGTTTCCTGACACGAGGTGA	AAACCAACAGGGCAATAGCA	NM_001105415.1
CRY2	GGGCCAGAGTCAGTGCTTG	ACCTTAGGGAAGAGACCCATCAT	NM_001289786.1
BMAL1	GCCAGAATGACCTGATTGGT	GCCGTGCTCCAGAACATAAT	NM_001191170.1
CLOCK	GGATCCATGCTTCCTGGTAATG	TGACTGTGCAGTGATTTCTTTATGTT	NM_001289769.1
REV-ERBα	AACCTCCAGTTGTGTCAAGG	GTGATGACGCCACCTGTGT	NM_001078100.2
RORα	GGCTAGCATATTTCCCATCGAA	CAAAGCAGTTCTCTAGAGGTGGTA	NM_001192861.1
NCOR1	TCTCGTAACTATCCGGGTGGA	CTTCTAGGAAATCACCGGACG	NM_001193059.2
CREB1	TGCCCCTGGAGTTGTTATGG	CTCGTGCTGCTTCCCTGTTC	NM_174285.1
SIRT1	GGGCTTACAGGGCCTATCCA	TCACCAAACAGAAGGTTATCTCGG	NM_001192980.3
NR2F2	CGGAGGAACCTGAGCTACAC	CCTCTGCACGGCTTCCC	NM_174402.3
FBXW11	GTGATTTTATCTAGGGAAGTGCAGA	ATCCTCCATAACTGAAGTGTTCTGA	XM_010816695.2
TIMELESS	ACCCTTGAGCTTGCCTAATG	GCCTTCCTGGTGCAGACTTT	NM_001102071.2
CD36	GATGAGCTCCTTAAGCCATTCT	TGTCTGCCTCAAGTGCTGG	NM_001046239.1
LPL	ATGAACTGGATGGCGGATGA	CCCCAAGGCTGTATCCCAAG	NM_001075120.1
ACSL1	ACCAGTGGAACTACAGGCAA	GAGGCAAGAAAGAGATCAAAGTGT	NM_001076085.1
ACSS2	GAGGAGCTCAAGAAGCAGATTAGA	GTCCACACGCTTCTGGTTAC	NM_001105339.1
FABP3	AGACCACAGCAGATGACAGG	GCCATGGGTGAGTGTCAGAAT	NM_174313.2
FABP4	AGATGACAGGAAAGTCAAGAGCA	GACACATTCCAGCACCATCTT	NM_174314.2
ACACA	AAGACGGACAAGCAGACGTT	TCCACTTCCAAAAAGAACTCAGAGA	NM_174224.2

FAS	AAATGCCCACATGGCTGGTA	TTTTCCGTTTGCCAGGAGGA	U34794.1
SCD	CATGGCGTTCCAGAATGACG	AAGAAAAAGCCACGTCGGGA	NM_173959.4
FADS1	GGCTCGTGATTGACCGAAAG	CCACAAAGGGATCCGTGG	XM_024987473.1
GPAM	CTGAGGCTTGAAATGCTGGC	TCCCGAATCATGTGCTGTCC	NM_001012282.1
AGPAT6	GGCGTACTCATCCGCTACTG	GAACTCCTTGAACCTCCCGT	NM_001083669.1
LPIN1	TTGCATACAAAGGCAGCCAC	AGTTCATGGTCTGAGCCTCG	NM_001206156.2
DGAT1	AACCTGACCTACCGCGATCT	GGGGGAAGTTGAGCTCGTAG	NM_174693.2
ADFP	GCGTCTGCTGGCTGATTTCT	TGTAAGCCGAGGAGACCAGA	NM_173980.2
BTN1A1	CATCTGAAGGTGGCTGCTCT	CACTGTACTTGGGGCTCTGG	NM_174508.2
XDH	ACTGGGCAAGGACTCGAAAG	CCATTGGGCACCTCTTGGAA	NM_173972.2
INSIG1	GGCCTCTGGTGGACATTTGA	GTCTGGGGACGTGTACTGAT	NM_001077909.1
SCAP	GGCACCTCATGGACATC	ATGCGGGTGAGGCAGTC	NM_001101889.1
SREBF1	ACGCCATCGAGAAACGCTAC	GTGCGCAGACTCAGGTTCTC	NM_001113302.1
SREBF2	ATGCACAAGTCTGGCGTTCT	ATGCCCTTCAGGAGTTTGCTC	NM_001205600.2
C/EBP	TGGAGCTGACCAGTGACAAT	AGTTCGCGGCTCAGTTGTTC	NM_176784.2
PPARA	CCAACAACAACCCGCCTTTC	CTCCTTGTTCTGGATGCCGT	NM_001034036.1
PPARG	ATTATTCTCAGTGGAGACCGCC	CAAGGCTTGCAGCAGATTGT	NM_181024.2
PPARD	GAGACAGCCTTGTGTGGTGT	AGTTCCCGTCAGCCTCTTTG	NM_001083636.1
LXRα	CCTGTCTCGCTCAGTTTGGG	AAGGGACTGTCCGGTGC	NM_001014861.1
ACTB	CCGCAACCAGTTCGCCAT	CCACGATGGACGGGAAGAC	NM_173979.3
GAPDH	AGGTCGGAGTGAACGGATTC	ATGGCGACGATGTCCACTTT	NM_001034034.2
UXT	TGAGCGACTCCAGGAAGCTA	GGGACCACTGTGTCAACGAA	NM_001037471.2

Table S2. Antibodies used for WB and IF

Antibody	Cat. No.	Source	Molecular Weight (kDa)	Dilution
PER2	R389106	Zen-Bioscience Co., Ltd.	137	1:600
FABP3	R381985	Zen-Bioscience Co., Ltd.	15	1:800
ACC	381131	Zen-Bioscience Co., Ltd.	266	1:600
SCD	R25675	Zen-Bioscience Co., Ltd.	42	1:800
LPIN1	R383104	Zen-Bioscience Co., Ltd.	99	1:800
DGAT1	R389103	Zen-Bioscience Co., Ltd.	55	1:800
ADRP	R381796	Zen-Bioscience Co., Ltd.	48	1:1000
SREBP1	347061	Zen-Bioscience Co., Ltd.	122, 65	1:600
PPARG	340844	Zen-Bioscience Co., Ltd.	58	1:1000
β-Actin	R23613	Zen-Bioscience Co., Ltd.	42	1:6000
goat anti-rabbit IgG	511203	Zen-Bioscience Co., Ltd.		1:6000
SREBP1	PA1-337	Thermo Fisher Scientific		1:300
goat anti-rabbit IgG	A-11078	Thermo Fisher Scientific		1:2000

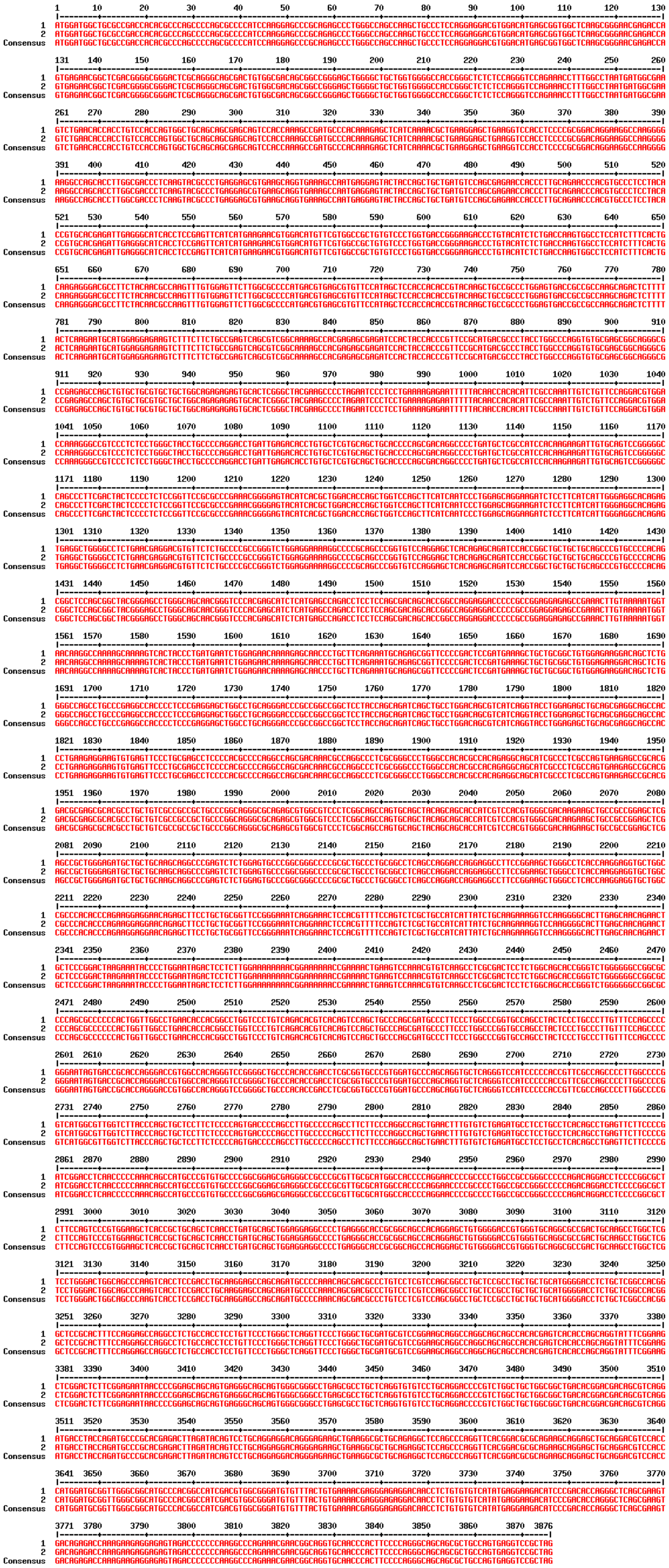

Figure S1. Genetic verification of eukaryotic expression plasmid construction. DNA sequence comparison was performed using SnapGene Viewer 5.2. *PER2* sequences of constructed plasmid consistent with the sequences posted on GenBank (https://www.ncbi.nlm.nih.gov/nucleotide/).
